# Suppressing the Chills: Effects of Musical Manipulation on the Chills Response

**DOI:** 10.3389/fpsyg.2018.02046

**Published:** 2018-10-29

**Authors:** Scott Bannister, Tuomas Eerola

**Affiliations:** Department of Music, Durham University, Durham, United Kingdom

**Keywords:** chills, continuous response, emotion, perception, psychophysiology

## Abstract

Research on musical chills has linked the response to multiple musical features; however, there exists no study that has attempted to manipulate musical stimuli to enable causal inferences, meaning current understanding is based mainly on correlational evidence. In the current study, participants who regularly experience chills (*N* = 24) listened to an original and manipulated version of three pieces reported to elicit chills in a previous survey. Predefined chills sections were removed to create manipulated conditions. The effects of these manipulations on the chills response were assessed through continuous self-reports, and skin conductance measurements. Results show that chills were significantly less frequent following stimulus manipulation across all three pieces. Continuous measurements of chills intensity were significantly higher in the chills sections compared with control sections in the pieces; similar patterns were found for phasic skin conductance, although some differences emerged. Continuous measurements also correlated with psychoacoustic features such as loudness, brightness and roughness in two of the three pieces. Findings are discussed in terms of understanding structural and acoustic features and chills experiences within their local music contexts, the necessity of experimental approaches to musical chills, and the possibility of different features activating different underlying mechanisms.

## 1. Introduction

Music has the remarkable capacity to affect listeners in several ways; music can elicit emotional responses in different people (Scherer and Zentner, 2001; Juslin and Västfjäll, 2008; Lundqvist et al., 2009), and can elicit physiological and bodily changes, such as increases in heart and respiration rate (Etzel et al., 2006; Gomez and Danuser, 2007), skin conductance (Khalfa et al., 2002), facial muscle activity (Krumhansl, 1997), and neurophysiological activity (Menon and Levitin, 2005; Salimpoor et al., 2011). Sometimes, music can elicit particularly strong emotional experiences (Gabrielsson and Lindstrom Wik, 2003; Lamont, 2011), resulting in changes of perspective and lifestyle (Schäfer et al., 2014). A specific emotional experience that has received attention in the field of music and emotion research is that of musical chills, thrills or frisson (Harrison and Loui, 2014). The experience, labeled in this context as chills, refers to a subjective feeling of emotion accompanied by gooseflesh, shivers or tingling sensations. Although little is currently known regarding the emotional characteristics of the response, it is suggested that chills are an indication of peak moments of pleasure (Blood and Zatorre, 2001; Salimpoor et al., 2009, 2011), and strong emotional experiences (Panzarella, 1980; Gabrielsson, 2011). The response has been linked to the mixed emotional state of being moved (Benedek and Kaernbach, 2011; Menninghaus et al., 2015; Wassiliwizky et al., 2015; Schubert et al., 2016), Kama Muta as elicited by communal sharing relationships (Fiske et al., 2017), feelings of awe (Keltner and Haidt, 2003; Konečni, 2005), fear responses (Huron, 2006), and empathic concern (Zickfeld et al., 2017). Chills have also been associated with aspects of the individual listener, such as the personality trait of openness to experience (McCrae, 2007; Nusbaum and Silvia, 2011; Nusbaum et al., 2014; Colver and El-Alayli, 2016), social contexts (Sutherland et al., 2009; Egermann et al., 2011), and familiarity with a piece of music (Panksepp, 1995; Rickard, 2004; Benedek and Kaernbach, 2011). Additionally, whilst there are concerns that gooseflesh and shivers may independently reflect different experiences (Maruskin et al., 2012), chills have been approached quite broadly in previous research, as a relatively successful index of strong emotional experiences in laboratory settings. Chills can be elicited by different kinds of stimuli, such as literature, visual art or film (Goldstein, 1980; Wassiliwizky et al., 2017), and the response has often been studied in the context of music, having been linked to various aspects of the music, the listener, and the listening situation. Existing research has placed an emphasis on the correlations between specific musical features and the chills response, a useful but limited approach that fails to test the relationships identified; this central shortcoming aims to be explored and addressed in the current study.

### 1.1. Chills and musical features

In one of the earliest studies to assess links between musical features and bodily responses whilst listening, Sloboda (1991) asked participants to recall experiences of bodily activity (e.g., heart rate change, laughter), to report the piece of music involved, and to identify as accurately as possible the specific moment in the piece that elicited the response. Interestingly, shivers, tears and feeling a lump in the throat were some of the most frequently reported bodily reactions to music; further links were found between each reaction and musical features, with shivers being associated with new or unprepared harmony, and changes in texture and dynamics. Panksepp (1995) documented a series of experiments that focused explicitly on the chills response. Participants listened to a collection of sad and happy pieces of music, and held their hand up to indicate the experience of a chill; from this, the frequency of chills across participants was documented in relation to temporal positions within a piece of music, suggesting that many chills experiences occurred during a crescendo in a popular piece of music from that time by Pink Floyd. Whilst findings for this piece were quite clear, others resulted in varied responses; either chills were distributed more evenly across a piece, or were linked to musical moments that contained no notable features distinct from the local contexts in the piece.

More recently, a series of advanced studies have been carried out on the associations between chills and musical features, utilizing skin conductance, a general indicator of emotional arousal (Khalfa et al., 2002; Carlson, 2012). Rickard (2004) assessed whether strong emotional experiences were characterized by higher levels of physiological arousal, reporting that when participants listened to emotionally intense music, skin conductance levels increased, as did the frequency of chills experiences. Craig (2005) also collected skin conductance data from participants, and found that when chills were subjectively reported by participants, skin conductance levels increased. Following the apparent association between skin conductance and chills, numerous studies have since utilised the measurement as an effort to verify the experience (Grewe et al., 2007; Guhn et al., 2007; Grewe et al., 2009; Egermann et al., 2011; Laeng et al., 2016). More recent correlational work developed further links between chills and specific musical features (Grewe et al., 2007; Guhn et al., 2007); interestingly, previous findings from Sloboda (1991) and Panksepp (1995) were supported through results linking chills to dynamic changes (Grewe et al., 2007). Some novel findings were reported however, with specific features such as the entrance of new voices or instruments (Grewe et al., 2007), and interactions between solo and accompaniment instruments (Guhn et al., 2007) correlating with chills. Furthermore, a psychoacoustic analysis was performed in accordance with the moments when chills frequently occurred, with conclusions suggesting that an increase in loudness and sharpness (linked to high frequency content of a sound) was found in the music during numerous chills responses (Grewe et al., 2007), and that chills passages were sometimes characterized by an expansion in the overall frequency range (Guhn et al., 2007); elsewhere, chills were further linked to a rise in auditory roughness and decreased tone-to-noise ratio (Nagel et al., 2008). However, it is important to note that changes in psychoacoustic parameters may be naturally correlated to other musical events such as the entrance of new instruments, or solo and accompaniment interactions.

The reviewed literature on musical chills and corresponding musical features suggests that the response may often be elicited by dynamic changes and crescendos. These musical characteristics might also be contextualized in terms of mechanisms of anticipation, expectancy and fear. Gooseflesh has been observed as a response in some mammals to perceived threat in the immediate environment (Darwin, 1872), either as a signal to conspecifics, as intimidation to the perceived threat, or as a contraction of muscles that may prevent blood loss in the event of injury (IJzerman et al., 2015). With a high adaptive value ascribed to being able to predict events, the violation of one's expectations might activate immediate fear processes. This attentional response appears to be prevalent in “safe” aesthetic contexts such as music listening, with various effects of musical expectancy found in event-related potential studies (Besson and Faita, 1995; Koelsch et al., 2002; Magne et al., 2006), and emotional arousal research (Steinbeis et al., 2006). Huron (2006) suggests that when expectations are violated, the brain develops a quick worst-case scenario appraisal, resulting in chills; in line with James (1894), these chills are perceived, and then appraised alongside the music in an aesthetic context, possibly resulting in pleasure given the immediate contrast between an initial negative reaction, and more inherently positive appraisal within a “safe” context. In Juslin's framework (Juslin, 2013), musical expectancy (syntactical expectations) and brain stem reflexes, an involuntary reaction to potentially important events, are considered as mechanisms of music and emotion that interact with fear and a variety of aesthetic judgments made by the listener. Another interesting aspect related to fear may be that of auditory looming, and the ability of detecting or perceiving an object or stimulus as approaching (Ghazanfar et al., 2002); this may offer an explanation as to why slower crescendos and gradual builds in intensity can result in chills responses (Bannister, 2018). As well as expectancy violations, there is also some evidence of anticipatory mechanisms underlying chills during music listening, with Salimpoor et al. (2011) noting distinctions in dopaminergic activity during chills, compared to moments preceding recorded chills responses; elsewhere, a distinction has been suggested in physiological activity, between the chill response and what was termed a “pre-chill” reaction (Wassiliwizky et al., 2017), which may map neatly on to pre-outcome and post-outcome phases of Huron's ITPRA model of expectation (Huron, 2006), and general theories of musical tension and release (Lehne and Koelsch, 2015). From this, it is intuitive that specific musical features and their associations to chills may largely depend on effects of local, preceding musical contexts; this remains a largely unexplored factor and is an important issue, in that depending on the intra-musical context, a similar musical characteristic may be more effective in eliciting chills. Although the link between fear and chills is quite intuitive, fear as a central mechanism struggles to explain why there are many listeners who never experience chills, and why these responses are often not consistent inter- and intra-individually.

Alternatively, other theorists have suggested that aesthetic chills have strong social underpinnings (Benedek and Kaernbach, 2011; Schurtz et al., 2012; Wassiliwizky et al., 2015; Schubert et al., 2016; Wassiliwizky et al., 2017), linked to ideas of social separation and closeness (Panksepp, 1998; Panksepp and Bernatzky, 2002), and the state of being moved (Konečni, 2005; Schurtz et al., 2012; Hanich et al., 2014; Wassiliwizky et al., 2015). Being moved can be conceptualized as a mixed emotional experience, involving two main components of happiness and sadness (Menninghaus et al., 2015). This response might be elicited by stimuli or events with prevalent social themes, such as weddings, births, funerals, and other significant events that invite empathic processes such as natural disasters (Kuehnast et al., 2014). The link between being moved and chills is not clearly understood, but may derive from possible evolutionary associations between thermoregulatory and social processes in the brain. Panksepp (1998) suggested that social and thermoregulatory circuits were closely situated and linked to each other in the brain, possibly resulting in interactive effects between processes; this entanglement is more recently supported by the finding that feelings of social and physical warmth are accompanied by similar patterns of neurophysiological activity and reports of phenomenology (Inagaki and Eisenberger, 2013). The origins of this coupling in the human brain is not immediately clear, but may stem from a human inefficiency of self-thermoregulation, increasing the adaptive value of utilizing conspecifics and social groups to share body heat and better manage internal temperatures (IJzerman et al., 2015). In the context of chills as a response to social cues, dynamic changes and crescendos are not open to immediately intuitive explanations. Instead, the link between chills and social aspects may be reflected by other musical features, such as moments of musical union, where instrumentalists all start to play together and large groups start chanting (Bannister, 2018), or in the alternations and contrasts of solo and accompaniment in performance (Guhn et al., 2007). Therefore, it could be possible that chills can not only result from musical expectancy or reactionary mechanisms such as brain stem reflexes, but also from empathic processes, emotional contagion, and interpersonal rhythmic entrainment (Juslin et al., 2010). Furthermore, depending on underlying mechanisms activated, the phenomenological experience of chills may be quite distinct, leading to potential issues regarding the broad terminology currently used (see Maruskin et al., 2012). However, in the current musical context, there is almost no empirical research that has attempted to experimentally test the chills response in terms of manipulating musical features and stimuli; instead, most studies have taken a correlational approach, resulting in a lack of development regarding the theoretical foundations of aesthetic chills, and a limited understanding of causal processes underlying the experience.

### 1.2. Rationale for the current study

The current study aimed to provide the first empirical exploration into the effects of manipulating real musical stimuli on the chills response. With plans to remove previously identified sections in certain musical stimuli that elicit chills, the three main aims of the research were to: Firstly, test whether chills can be manipulated in empirical settings; secondly, assess whether chills are linked to specific features, qualities and sections in music; and finally, assess how removing chills sections affects the frequency and experience of chills. The main hypothesis of the study was that pre-identified chills sections in three musical stimuli would result in more chills experiences and stronger emotional responses compared to other notable moments in the piece that are either characterized by structural changes previously correlated with chills (Sloboda, 1991; Panksepp, 1995; Grewe et al., 2007), or by their psychoacoustic similarity to the chills eliciting section; additionally, it was expected that when removing the chills section to create manipulated versions of each piece, the frequency of chills experienced across listeners would be reduced.

## 2. Methods

### 2.1. Design

**Figure 1:**
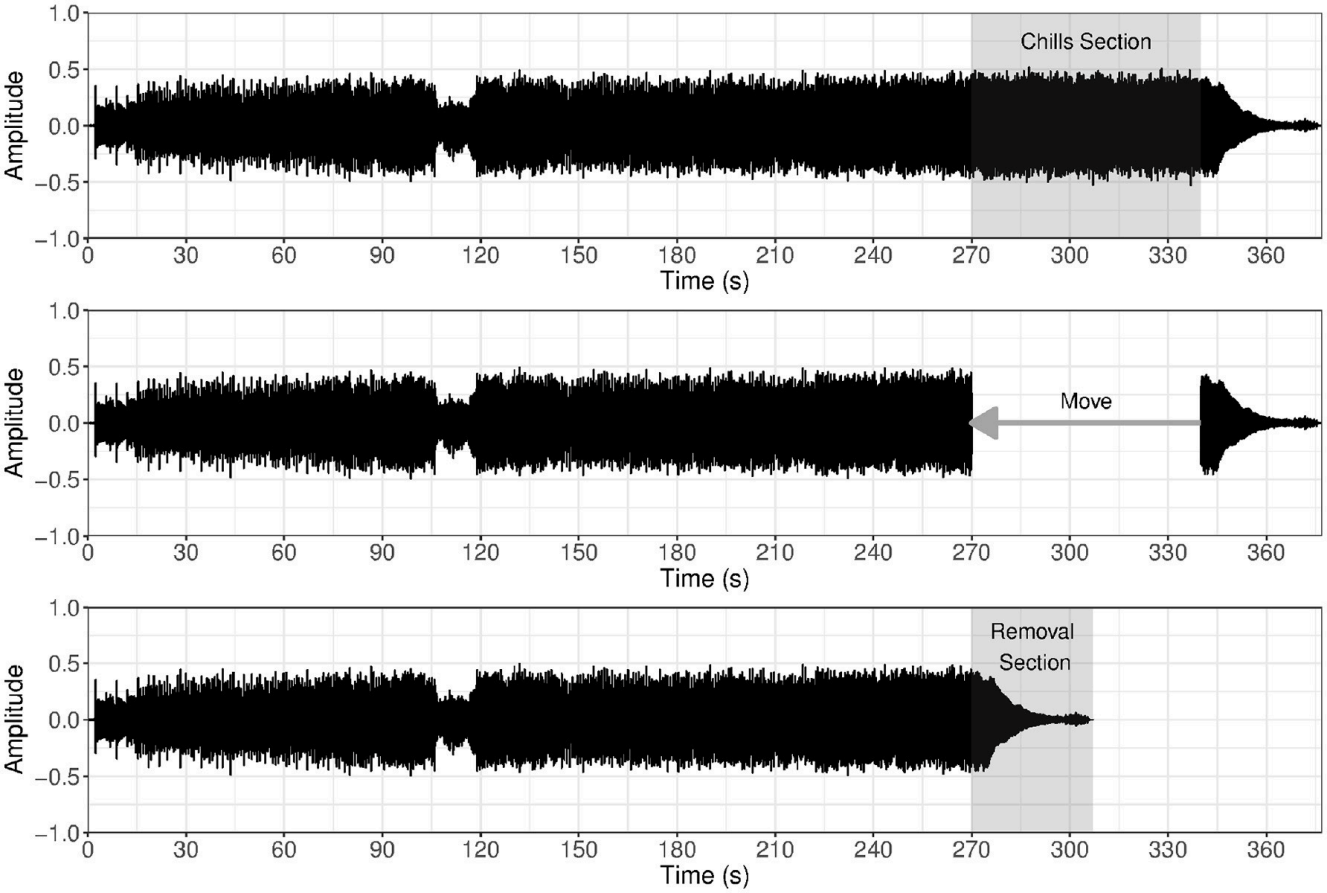
Example of audio editing procedure to create manipulated stimuli. First, the *chills section* is identified; second, the chills section is removed from the piece; thirdly, the music following the chills section is moved and spliced with the music that came before, resulting in what is termed the *removal section*. The figure depicts the piece *Glósóli* by Sigur Rós.

A listening experiment was carried out using three musical stimuli that were reported to elicit chills in listeners from a previous survey (Bannister, 2018); for a full list of chills pieces from this survey, see the corresponding online dataset (Bannister and Eerola, 2017). For each stimulus, there were two listening conditions corresponding to the original version and the manipulated version of the piece, resulting in a total of six listening conditions. To create the original and manipulated listening conditions, the musical stimuli were edited, by removing the *chills section* in its entirety from the piece, and then splicing together the music from before the chills section with the music that followed the end of the section; the characteristics of the preceding and subsequent musical sections allowed for the maintenance of logical and natural progressions in the manipulated versions (see Figure 1). These chills sections were determined through participant reports from the previous survey, combined with musicological analysis of the musical development and structure, to produce a meaningful demarcation of start and end points for the sections. To make the editing as non-disruptive as possible, sophisticated cross-fade techniques were applied to splicing points in the audio. In pilot testing, listeners unfamiliar with the pieces of music were under no suspicions that the manipulated versions of the music had been altered or modified. This method of manipulation has the advantage of maintaining high ecological validity, but comes at the cost of control over variables in the music, and higher fatigue for participants in listening to six stimuli of 5–8 min in length. All audio editing was completed in the Logic Pro software.

The central dependent variables included a self-report of chills experiences, skin conductance levels, and continuous measurements of chills intensity. The experiment followed a repeated-measures design, with participants listening to both versions of the three musical stimuli. To limit effects of repetition and fatigue, the stimulus presentation order was pseudo-randomized for each participant, such that no version of the same piece directly preceded or followed the other, and that presentation order was different for every participant. Additionally, the experiment was partitioned into two blocks of listening, with three musical stimuli in each; these blocks were separated by a short questionnaire, extending the break between listening blocks to roughly 10 min.

### 2.2. Participants

All participants were selected through a pre-screening process, which involved completing a questionnaire confirming that participants had experienced musical chills before, and that the response occurred relatively frequently (e.g., monthly). A total of 24 participants took part in the listening experiment. Of the sample, 17 were female, with a mean sample age of 25.2 (*SD* = 5.96, range 18–46). For the full descriptive statistics, please see the Supplementary Material.

### 2.3. Materials and measures

#### 2.3.1. Self-report items

For every piece that participants listened to, a series of rating scales were completed. Immediately after each piece, participants were asked to do the following: Firstly, confirm whether they experienced chills whilst listening (yes/no); secondly, rate how they felt across 11 emotional descriptors derived from the Geneva Emotional Music Scales (GEMS) on scales of 1–7 (Zentner et al., 2008); thirdly, rate the intensity of their emotions and how moved they were (Likert, 1–7); finally, complete an updated experimental version of the MecScale (Juslin et al., 2014, 2015) to assess the potential significance of underlying mechanisms in the chills experiences (Likert, 1–7). This instrument was updated, following some inconsistencies with the original version when compared to corresponding qualitative data in the previous survey. For the GEMS and MecScale descriptors, the order of presentation in the questionnaire was shuffled in the second block of listening to limit effects of fatigue and automaticity of responses. Finally, after each block of three stimuli, participants were asked to rate how much they enjoyed each piece, how familiar they were with the music (1–5, corresponding to statements of the degree of familiarity), and to describe their favorite moment in the piece. Finally, individual differences such as music sophistication were collected using a reduced version of the Goldsmith's Musical Sophistication Index (Müllensiefen et al., 2014), an instrument linked to aspects of aptitude and musical skill, but also to more broad interactions and engagement with music; musical preferences were captured using the short test of musical preferences (STOMP; Rentfrow and Gosling, 2003).

#### 2.3.2. Stimulus selection

The selection of the musical stimuli was informed by the previous survey on musical chills (Bannister, 2018). Three pieces were chosen as stimuli for the experiment in accordance with a set of criteria: Firstly, the piece of music needed to be highlighted by two or more participants in the survey; secondly, participants had to be able to determine and agree on a specific moment in the piece that elicited chills; thirdly, the piece needed to be suitable for manipulation and audio-editing processes (smooth, natural progression following manipulation); and finally, the stimulus would need to be of a usable duration (no longer than 9 min), to control for the length of the experiment and possible differences in results as a consequence of stimulus duration. Resulting from the selection process and criteria, the first piece selected was *Glósóli* by Sigur Rós. The piece can be described as rock or post-rock with Icelandic lyrics, and a focus on atmosphere and texture developed by electric guitars. Structurally the piece is quite simple, utilizing two different chord progressions and a long build-up to what has been specified previously as the “chills section”, namely a crescendo and dynamic climax of distorted guitars and loud drums, starting at 4:34. The second stimulus selected for the experiment was *Jupiter* by Gustav Holst. The piece belongs to a larger orchestral suite, and is instrumental, upbeat and energetic. The chills section identified in this piece was a slower, thematic string progression in the middle of the piece, beginning at 3:09 and ending at 4:55. The melody has elsewhere been adapted to lyrics, and used in international sporting events, and so may be well-known by listeners, regardless of familiarity with the overall piece. The final stimulus selected from the survey was *Ancestral* by Steven Wilson, an electronic progressive rock piece characterised by a large shift from electronic ambience to more traditional hard rock instrumentation. The original piece is over 13 min in length, and was shortened to 6 min, justified by no reports in the previous survey of chills occurring in the second half of the piece; care was taken to make sure the music sounded complete, and that the shorter version did not end abruptly. The chills section reported for this piece was during a guitar solo toward the end of the piece, starting at 4:02 and ending at 5:02. A final aspect to note is that Ancestral is the only piece to contain English lyrics, which may affect the listening experience. Both Glósóli and Ancestral were assessed *a priori* to be unfamiliar pieces of music by the authors, further confirmed by ten other listeners, whereas Jupiter was predicted to be more familiar; experiment results thoroughly support these assessments. Detailed time stamp information for all sections reported in this study can be found in the corresponding online dataset and documentation (Bannister and Eerola, 2017).

#### 2.3.3. Chills measurement

To capture the experience of musical chills, participants reported the intensity of their chills whilst listening using a continuous rating paradigm, and their skin conductance was recorded. Skin conductance was preferred as it is a non-intrusive procedure, which is important when considering strong emotions in laboratory settings; additionally, the measure has been frequently utilized as a reliable indicator of chills in previous research (Craig, 2005; Grewe et al., 2007; Benedek and Kaernbach, 2011). Skin conductance data were captured with two electrodes (Ag/AgCL) attached to the distal phalanx of the index and middle fingers of the non-dominant hand with the NeXus-10 MKII and BioTrace software. As skin conductance is only indicative of emotional arousal, continuous subjective measurements were also used. For these continuous measurements, data were collected with an analog slider, moved upwards to indicate higher levels of chills intensity, or vice-versa; slider movement changed amplitude values of a monitored and recorded sine wave, which was preferred over a rating scale, as it was deemed to be less distracting, and a simpler task. It was important to keep this task as simple as possible, as concurrent tasks during listening may have the capacity to affect physiological and emotional responses (Jäncke et al., 2018). Chills are normally reported by participants, either by raising their hand to indicate the response (Panksepp, 1995; Craig, 2005) or by pressing a button (Grewe et al., 2007; Salimpoor et al., 2009). Whilst convenient for analysis and assessing the frequency of chills within a piece, it is most likely that chills are subjectively experienced differently across individuals, and the dichotomous distinction between having chills or not may not accommodate these differences in recognition or intensity. Therefore, the exploratory use of continuous measurements over typical button press paradigms was motivated by the likelihood that chills are not only experienced differently across listeners, but that they can also occur within people at varying intensities (Craig, 2005).

### 2.4. Procedure

Participants were each tested separately and were asked to familiarise themselves with the experiment by reading an information sheet provided (see the Supplementary Material for a procedural outline, and information on the pseudorandomization of stimulus presentation order). Informed consent was obtained through a signature and confirmation that the subject understood the procedure of the experiment. Before the listening exercise began, electrodes were attached to the non-writing hand of participants. Finally, before initiating the main procedure, participants listened to a short excerpt of instrumental guitar music, and were asked to become familiar with the analog slider for the continuous measurements. In the first block of listening participants listened to either an original or manipulated version of the three stimuli. After each piece had finished, participants completed the rating scales provided. When ready for the next piece to start, participants could communicate to the investigator in a separate room by using a microphone set up close by. Once participants had listened to the three pieces, they were asked to complete a set of questions, including distractors and others concerning musical preferences and demographics. In the second block of listening, the remaining versions of the three musical stimuli were listened to, following the exact same procedure as the first block. To conclude the experiment, participants responded to questions regarding musical sophistication, and the frequency of chills experiences in everyday life. Participants were reimbursed $5 for taking part. The experiment lasted approximately 1 h, and was approved by the local University Ethics Committee. All self-report instruments and data, physiological data, continuous measurement data, and stimulus information can be openly accessed on Harvard Dataverse (Bannister and Eerola, 2017).

### 2.5. Data analysis

All data were processed fully or partially in the R environment (https://cran.r-project.org), with Bonferroni corrections applied for all multiple comparisons; data that were not independent were analyzed with repeated-measures ANOVA tests and mixed effects models. Where parametric tests were utilized, residual plots were assessed for any clear deviations from normality or homoscedasticity. For mixed effects models, marginal *r*^2^ was calculated as an indicator of effect size following calculations from Nakagawa and Schielzeth (2013), and this was performed with the “*MuMIn*” R package (Barton, 2018); for ANOVA tests partial *eta*^2^ was calculated. For musical sophistication, genre preferences and underlying mechanisms, data were aggregated in accordance with distinct factor structures identified in previous studies (Rentfrow and Gosling, 2003; Müllensiefen et al., 2014). Skin conductance data display a negative trending behavior (gradual and linear decrease) over longer periods of time, which was corrected by applying a simple linear model to the data (de-trending). Skin conductance data is also comprised of tonic, baseline biological activity, and phasic event-related activity (Boucsein, 2012), and the two underlying signals need to be separated and identified to accurately analyse physiological patterns. To achieve this, the Ledalab toolbox in Matlab was utilised (Benedek and Kaernbach, 2010). Firstly, all raw data imported into Ledalab were pre-processed, by manually removing artifacts (e.g., sharp peaks from physical movement or biological variation), and by applying a smoothing Butterworth low-pass filter (order = 1, lower cut-off = 5). Next, the continuous decomposition analysis (CDA; Benedek and Kaernbach, 2010) method was applied to the data, to decompose the raw skin conductance measurements into tonic and phasic signals; this method underwent two optimization processes that estimates a best fit of the decomposed signal to the original (indicated by tau values), and was performed with the significant peak value set at 0.001. Following CDA, skin conductance data were normalised and baseline corrected within each participant to control for individual differences in the skin conductance response (Khalfa et al., 2002).

In the current analysis, the phasic skin conductance response (SCR) was a central focus, in terms of average amplitude levels for three regions of interest, namely the “*chills section*” in the piece, three “*control sections*”, and the “*removal section*” in the manipulated version of the piece; these regions were also utilised for continuous measurement analysis, with both sets of data log-transformed before comparisons to correct for non-normal data distributions. The chills sections for each piece were the predetermined moments found in the previous survey that elicit chills; the control sections were three shorter moments in the music that did not overlap with the chills sections, serving as a within-piece comparison; finally, the removal sections in the manipulated condition referred to the same moment in the music where the chills section would have started, but instead was comprised of musical progressions that would have followed the chills section before editing (see Figure 1), providing a test of direct effects of musical manipulation. For Jupiter, the chills section was 107 s in length, compared to a removal section of equal length, and three control sections of 36, 36, and 35 s, respectively. However, for Glósóli and Ancestral, chills sections were limited to the first 36 s, compared to a removal section of equal length, and three control sections of 12 s each; this duration limit was a result of manipulated versions being too short, such that the piece would not continue beyond the duration of the original chills section, making comparisons between removal sections and full duration chills sections impossible.

To strengthen the analysis, three shorter *musical-control* sections in each piece were first specifically selected to contain notable musical features of their own linked to chills, such as dynamic changes, entrances of new instruments, and repetitions of a theme (Sloboda, 1991; Panksepp, 1995; Grewe et al., 2007). In addition to these musical-control sections, a second set of *acoustic-control* sections were established based on psychoacoustic similarity to the chills section in question; utilizing MIR Toolbox (Lartillot et al., 2008), musical content similarity was calculated by comparing Euclidean distances between the chills section and every other part of the piece using mel-frequency cepstrum coefficients (MFCCs), cepstrum profiles, and spectral content (range 80–3,000 Hz), with a window size corresponding to the duration of the chills section. For Glósóli and Ancestral, frame lengths for comparison were 12 s, and for Jupiter frames were 35 s, with each iteration shifting the section of comparison forward by one second. From these indices of similarity, acoustic-control sections were determined through peak detection in the acoustic similarity values within the piece, focussing on points of peak convergence across the various psychoacoustic measures; peaks were defined as similarity values higher than the previous and successive values by a magnitude of 10 percent (or higher) of the range between maximum and minimum similarity values in the analysis. This chills vs. acoustic-control section comparison thus involved a novel set of three control sections that most resembled the chills sections in psychoacoustic terms.

Further psychoacoustic features of the musical stimuli were identified and analyzed with MIR Toolbox, following procedures previously documented by Eerola (2011). Low-level features included RMS values, brightness and roughness, whereas some other higher-level features were explored such as event density, key clarity and pulse clarity. As reviewed earlier, some of the low-level features such as loudness and roughness have previously been correlated with musical chills (Grewe et al., 2007; Nagel et al., 2008). The high-level features were an exploratory effort to capture changes in textural density (such as the number of onsets within each short segment in event density), tonal changes (key clarity indexes how perceptually well-defined the key is at each moment in time), and how salient the underlying pulse of the music is (pulse clarity). These features were downsampled to the same rate as the continuous ratings and skin conductance recordings (32 Hz), to allow for correlations with SCR and continuous measurement data.

## 3. Results

### 3.1. Frequency of chills

Across six listening conditions, three original and three manipulated, self-reports of chills experiences were collated, with the distribution assessed through the McNemar's test due to a repeated-measures design; each quadrant of the contingency table addressed whether participants had chills in just the original condition, just the manipulated condition, both conditions, or no conditions. Overall effects of the experimental manipulation on the frequency of chills show a marginally significant difference in distribution of chills depending on listening conditions (*x*^2^ = 3.85, *p* = 0.049, ϕ = 0.23); this is reflective of a consistent nominal but non-significant pattern found for each piece, with all original conditions resulting in a higher frequency of chills responses across listeners. For Glósóli, participants reported in total 10 experiences of chills in the original condition, and 7 in the manipulated version (*x*^2^ = 1.00, *p* = 0.31, *ϕ* = 0.20); for Jupiter, 10 episodes of chills were reported for the original condition, and 8 for the manipulated version (*x*^2^ = 0.50, *p* = 0.47, *ϕ* = 0.14); finally, participants reported 10 experiences of chills in the original version of Ancestral, and 6 in the manipulated condition (*x*^2^ = 1.28, *p* = 0.25, *ϕ* = 0.23).

### 3.2. Self-report data

#### 3.2.1. Emotions

**Figure 2:**
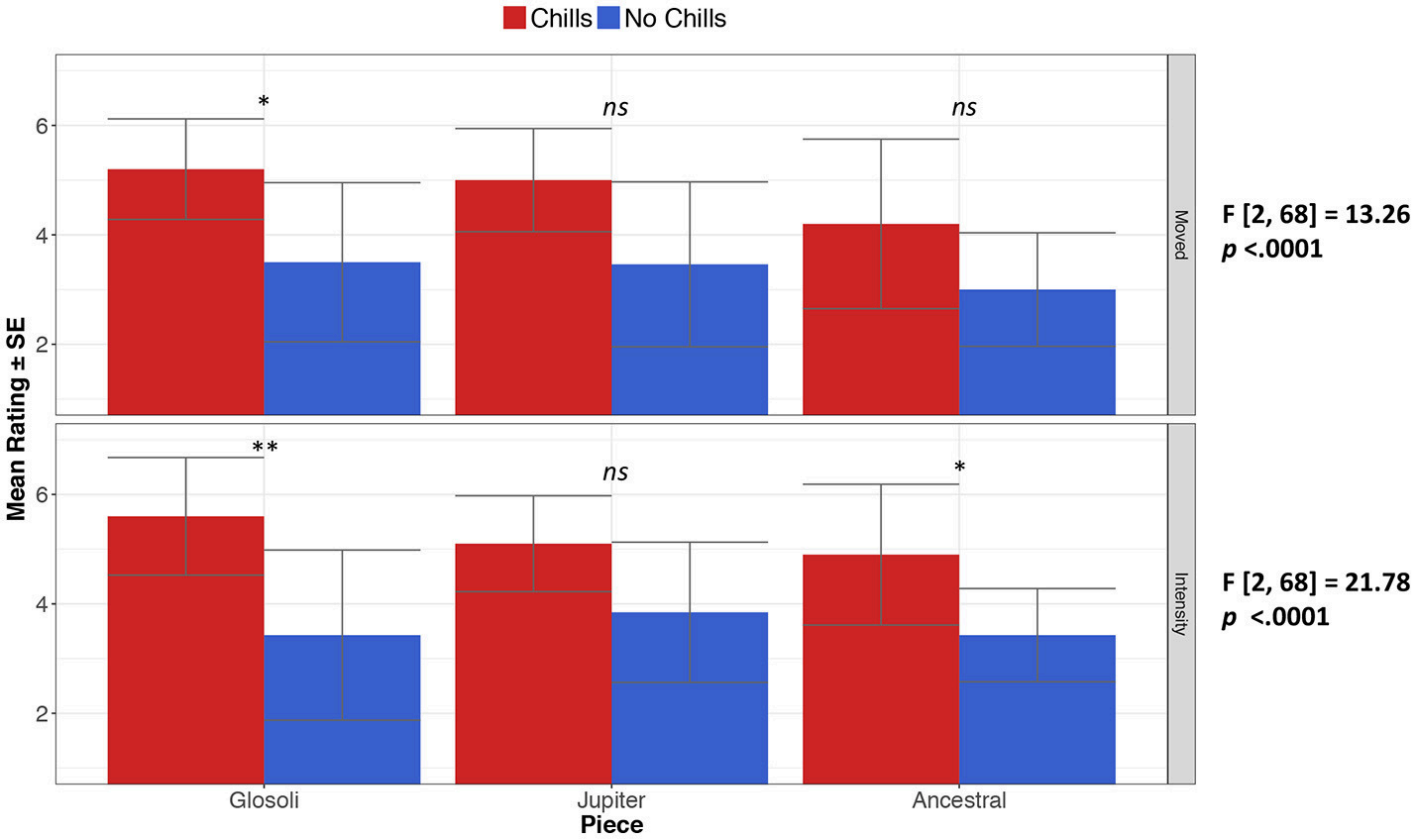
Mean ratings of being moved and emotional intensity for original conditions, depending on whether chills were experienced during listening (*p < 0.05, **p < 0.01); error bars indicate standard errors.

After each piece, participants responded to scales regarding feeling moved and emotional intensity (see Figure 2). The differences between these measurements were then assessed depending on whether participants had reported chills or not, utilizing a one-way MANOVA test with being moved and emotional intensity as two dependent variables, and self-reports of chills experiences (yes/no) as the independent variable. In the original conditions, the presence or absence of chills experiences had a significant effect on being moved and intensity ratings [*F*_(2, 68)_ = 16.88, *p* = < 0.0001, partial *eta*^2^ = 0.33], with further ANOVA tests confirming the effect for being moved [*F*_(2, 68)_ = 13.26, *p* = < 0.0001, partial *eta*^2^ = 0.25], and emotional intensity [*F*_(2, 68)_ = 21.78, *p* = < 0.0001, partial *eta*^2^ = 0.33]; further corrected comparisons showed that being moved ratings were significantly higher when chills were experienced in Glósóli (*t* = −3.25, *p* = 0.02, *d* = 1.34), but not in Jupiter or Ancestral. For emotional intensity, corrected comparisons showed that ratings were significantly higher when chills were experienced in Glósóli (*t* = −3.80, *p* = 0.005, *d* = 1.57) and Ancestral (*t* = −3.37, *p* = 0.01, *d* = 1.39), but not for Jupiter. Interestingly, the same one-way MANOVA revealed similar results in the manipulated conditions [*F*_(2, 67)_ = 5.29, *p* = 0.007, partial *eta*^2^ = 0.13]. To explore whether chills were more moving or intense in the original stimuli, ratings were compared across experiences of chills in original and manipulated conditions; a difference approaching significance between conditions was found for being moved and emotional intensity ratings in chills experiences [*F*_(2, 48)_ = 2.67, *p* = 0.07]; notably, chills were rated as more intense in the original conditions [*F*_(2, 48)_ = 10.22, *p* = 0.0004, partial *eta*^2^ = 0.11], but not more moving.

Finally, to assess how removing specific chills sections affected more general feelings during listening, emotional descriptors and ratings for underlying mechanisms of musical emotion were also assessed. A total of 11 descriptors were derived from the GEMS model, with Glósóli experiences rated as relaxing, calm and inspiring, Jupiter as happy, energetic and powerful, and Ancestral as sad, tense and powerful; these descriptors suggest that whilst chills are experienced at similar rates across the three pieces, the emotional qualities may vary. No ratings of emotion differed significantly between listening conditions for each piece, suggesting that the overall emotional experience was maintained even after removing chills sections in the music. Similar patterns were also found in ratings of underlying mechanisms: for all pieces, rhythmic entrainment and emotional contagion mechanisms received high scores, and no significant differences were found for any mechanism across original and manipulated conditions.

#### 3.2.2. Musical preferences, sophistication, and familiarity

In terms of musical preferences and chills, some differences were found across the four main factors of the STOMP, labeled *reflective and complex* (*M* = 4.69, *SD* = 1.18), *intense and rebellious* (*M* = 4.68, *SD* = 1.60), *upbeat and conventional* (*M* = 4.13, *SD* = 1.16), and *energetic and rhythmic* (*M* = 4.00, *SD* = 1.33). In a one-way MANOVA with the experience of chills (yes/no) as the independent variable and four preference factors as dependent variables, a significant effect was found for Glósóli [*F*_(4, 43)_ = 3.67, *p* = 0.011, partial *eta*^2^ = 0.25] and Jupiter [*F*_(4, 42)_ = 2.62, *p* = 0.048, partial *eta*^2^ = 0.19], but not for Ancestral. Further corrected comparisons revealed greater preferences for reflective and complex genres for participants who experienced chills in Glósóli conditions (*t* = 2.98, *p* = 0.018, partial *eta*^2^ = 0.16), and Jupiter conditions (*t* = −2.80, *p* = 0.029, partial *eta*^2^ = 0.14), when compared with non-chill responders.

To assess whether participants scoring higher on musical sophistication measures experienced more chills during the experiment (*M* = 31.12, *SD* = 8.65, possible range = 7–42), listeners were separated into a high chills group (experienced chills at least once in 4–6 of the pieces), a low chills group (experienced chills at least once in 1–3 of the pieces), and a no chills group; a one-way ANOVA indicated no significant differences for musical sophistication scores as dependent on the number of chills experienced in the experiment [*F*_(2, 18)_ = 0.78, *p* = 0.47].

In terms of familiarity, Jupiter was the most familiar with a mean rating of 3.83 (*SD* = 1.46), whilst both Glósóli (*M* = 1.60, *SD* = 1) and Ancestral (*M* = 1.50, *SD* = 0.97) were generally unfamiliar to participants; ratings of familiarity did not differ across chills and non-chills listening experiences. Interestingly, when comparing across original and manipulated versions, a significant difference in familiarity ratings was found for Jupiter (*t* = 5.77, *p* < 0.0001, partial *eta*^2^ = 0.60), with participants less familiar with the manipulated version.

### 3.3. Skin conductance

For skin conductance, mean phasic SCR activity levels were analyzed across chills sections, musical-control and acoustic-control sections, and removal sections for each piece. Phasic SCR was preferred over tonic activity as it better represents event-related physiological responses (Boucsein, 2012). For visualizations of the raw mean phasic SCR across participants, see the Supplementary Material. Due to occasional hardware issues, usable data were not collected from all 24 participants (Glósóli: original *N* = 19, manipulated *N* = 21; Jupiter: original *N* = 20, manipulated *N* = 21; Ancestral: original *N* = 21, manipulated *N* = 20).

**Figure 3:**
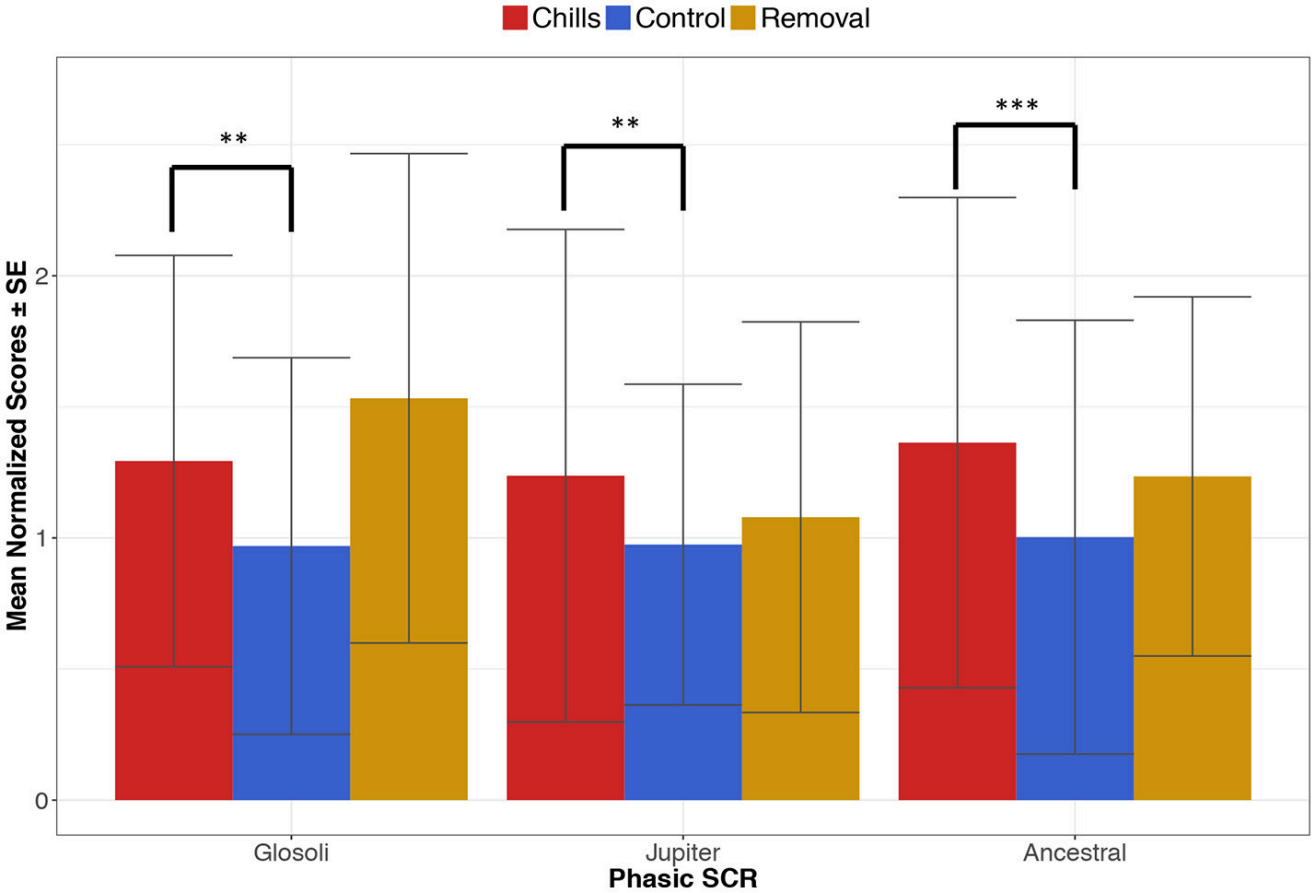
Average phasic SCR during chills, musical-control and removal sections within each piece (**p < 0.01, ***p < 0.001); error bars indicate standard errors.

The first analysis included musical-control sections, containing structural features previously linked to chills. A mixed-effects linear model was applied to the SCR data using the “*nlme*” package for R (Pinheiro et al., 2017), comparing means across three conditions (chills, musical-control, removal) with a random effect to compensate for participants contributing to data in every condition and piece; this method was chosen over a standard repeated-measures ANOVA because of a higher level of flexibility in planned *post-hoc* comparisons. Results for Glósóli showed a significant effect of condition on SCR [*F*_(3, 56)_ = 9.14, *p* < 0.0001, *r*^2^ = 0.25], with planned contrasts showing that SCR in the chills section was significantly higher than musical-control sections (*z* = 2.81, *p* = 0.004), but not statistically different from the removal section (*z* = −1.52, *p* = 0.12). Results from Jupiter also show a significant effect of condition on SCR values [*F*_(3, 58)_ = 3.65, *p* = 0.017, *r*^2^ = 0.11], with SCR in the chills section significantly higher than the musical-control sections (*z* = 3.04, *p* = 0.002), and nominally, but not significantly, higher than the removal section (*z* = 1.77, *p* = 0.075). Finally, results for Ancestral showed a significant effect of condition on SCR values [*F*_(3, 58)_ = 5.52, *p* = 0.002, *r*^2^ = 0.16], with SCR higher in the chills section compared to the musical-control sections (*z* = 3.71, *p* = 0.0002), but not with the removal section (*z* = 0.73, *p* = 0.46). A visualization of these results is presented in Figure 3.

The same analysis procedure was carried out a second time, but this time with acoustic-control sections selected based on their psychoacoustic similarity to the chills section. The mixed-effects linear model again found significant effects of condition on SCR for all pieces [Glósóli: *F*_(3, 56)_ = 11.76, *p* < 0.0001, *r*^2^ = 0.30; Jupiter: *F*_(3, 58)_ = 4.02, *p* = 0.011, *r*^2^ = 0.11; Ancestral: *F*_(3, 58)_ = 10.45, *p* < 0.0001, *r*^2^ = 0.26], but this time only chills and acoustic-control sections were subject to planned comparisons; these comparisons supported results from the first analysis, with phasic SCR being significantly higher in chills sections when compared to acoustic-control sections, for all pieces (Glósóli: *z* = 2.99, *p* = 0.002; Jupiter: *z* = 3.31, *p* = 0.0009; Ancestral: *z* = 4.55, *p* < 0.0001). It is important to note that whilst phasic SCR was the main focus, the same analysis was carried out for tonic SCL data with very similar results.

### 3.4. Continuous measurements of chills intensity

A further validation of chills was the continuous measurements of chills intensity. The same analysis strategy was carried out as with skin conductance, comparing mean measurements across chills, control and removal sections in each piece; again, the analysis was completed twice, one including musical-control sections derived from musicological considerations, and the other including acoustic-control sections assessed to be comparable to chills sections in psychoacoustic terms. For the raw averaged continuous self-report data across participants, see the Supplementary Material. As some participants did not experience chills, usable data were not collected from all participants (Glósóli: *N* = 20; Jupiter: *N* = 21; Ancestral: *N* = 20).

**Figure 4:**
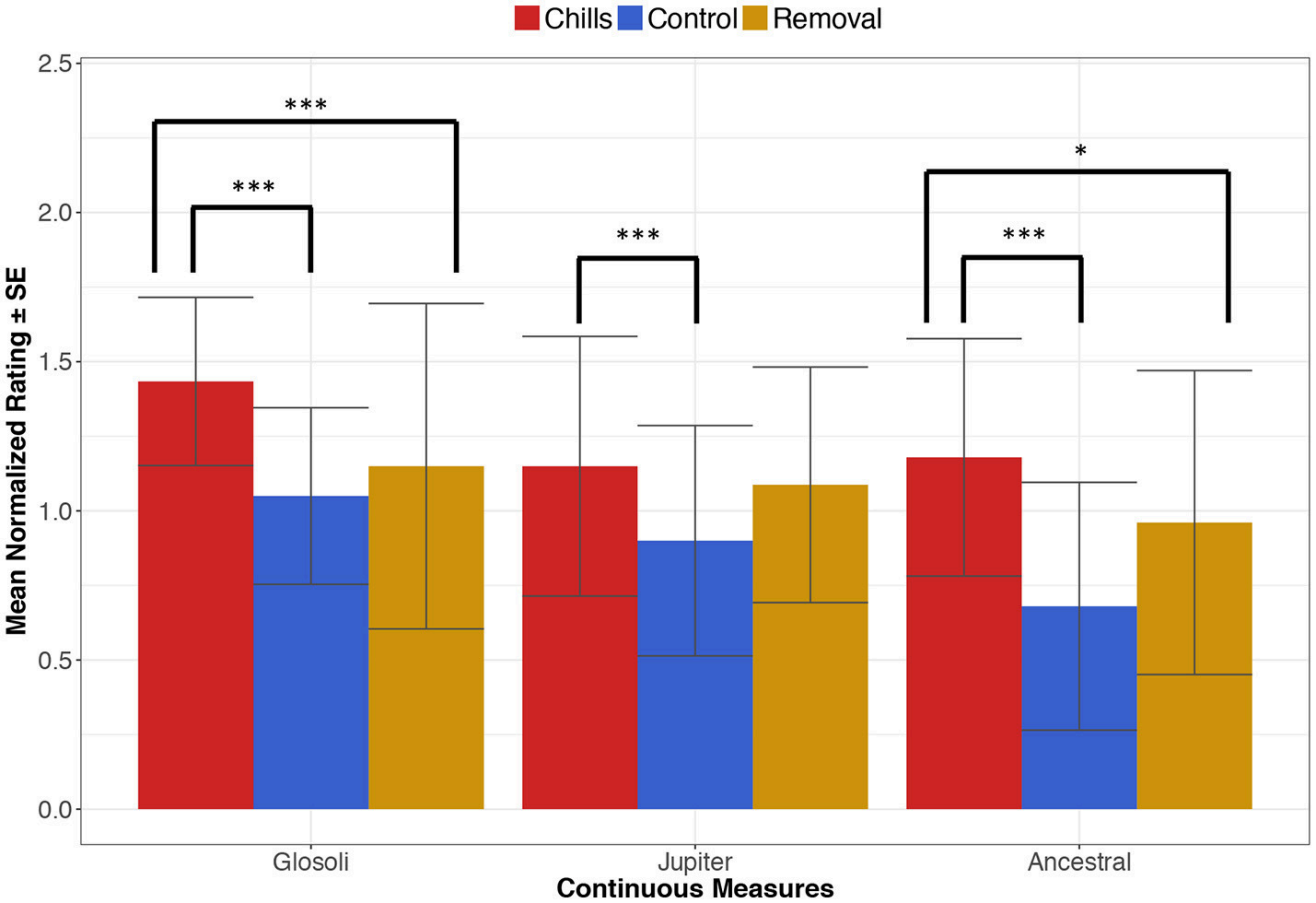
Mean continuous measurement ratings, comparing chills, control (musicological) and removal sections for each piece (*p < 0.05, ***p < 0.001); error bars indicate standard errors.

For the first analysis involving musical-control sections, a significant effect of condition on continuous measurements was found for Glósóli [*F*_(3, 57)_ = 10.79, *p* < 0.0001, *r*^2^ = 0.12], with *post-hoc* comparisons showing that continuous measurements were significantly higher in the chills section than the musical-control section (*z* = 5.41, *p* = < 0.0001) and the removal section (*z* = 4.00, *p* < 0.0001). For Jupiter, a significant effect of condition was found [*F*_(3, 60)_ = 6.34, *p* < 0.0001, *r*^2^ = 0.04]; planned contrasts revealed a significant difference between the higher ratings in the chills section and the lower ratings in the musical-control sections (*z* = 4.18, *p* < 0.0001), but no significant difference between chills and removal sections (*z* = 1.04, *p* = 0.29). Finally, results for Ancestral showed a significant effect of condition [*F*_(3, 57)_ = 10.75, *p* < 0.0001, *r*^2^ = 0.13], with *post-hoc* comparisons showing that continuous measurements in the chills section were significantly higher than both the musical-control sections (*z* = 5.52, *p* < 0.0001) and the removal section (*z* = 2.41, *p* = 0.015). A visualization of these results is presented in Figure 4.

For the second analysis involving acoustic-control sections, the mixed-effects linear model again found significant effects of condition on continuous measurements for all pieces [Glósóli: *F*_(3, 57)_ = 26.90, *p* < 0.0001, *r*^2^ = 0.22; Jupiter: *F*_(3, 60)_ = 4.72, *p* = 0.005, *r*^2^ = 0.03; Ancestral: *F*_(3, 57)_ = 23.57, *p* < 0.0001, *r*^2^ = 0.29]. In comparing chills and acoustic-control sections, results showed that continuous measurements were significantly higher in chills sections for all pieces (Glosoli: *z* = 8.14, *p* < 0.0001; Jupiter: *z* = 3.64, *p* = 0.0002; Ancestral: *z* = 7.24, *p* < 0.0001).

### 3.5. Monte carlo simulation approach

**Figure 5:**
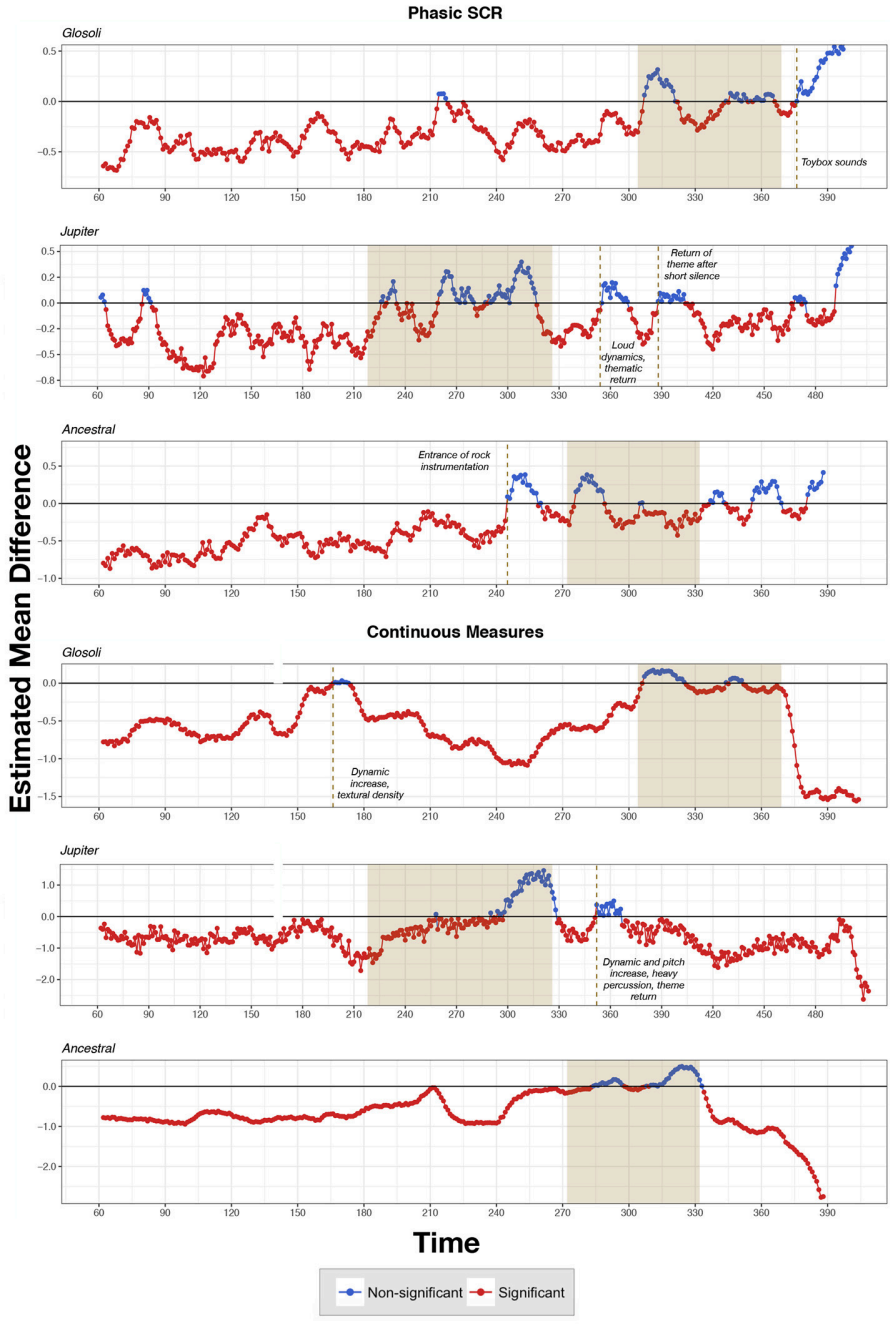
Estimated mean differences from the Monte Carlo simulations, comparing random 5 s control sections from any point in the piece, to random 5 s sections from within the shaded chills section. Dashed lines indicate notable structural moments in music linked to non-significant differences. Data points are visualized as significant when the estimated mean difference was below zero.

To eliminate any notion that the section comparisons reflect convenient points for analysis, and to further corroborate and support the results regarding skin conductance and continuous measurements across chills and control sections in the three pieces, a Monte Carlo Simulation process was utilized, with mixed-effects linear models fitted 10,000 times for each original piece; each iteration of the model would compare mean values of phasic SCR and continuous measurements across a randomly selected 5 s epoch extracted from the chills section, with a randomly selected epoch of equal duration from any moment in the piece (including the chills section). For every model, confidence intervals were extracted, with values averaged over 10,000 iterations. This approach was applied only to original versions of the stimuli to support control section selections, and validate the general emotional efficacy of chills sections. Results of multiple comparisons with phasic SCR showed that for Glósóli, average 95 percent confidence intervals for the mean differences between control and chills selections were −0.30 and −0.24; results were similar for Jupiter (mean 95 percent CI = −0.21, −0.15) and Ancestral (mean 95 percent CI = −0.37, −0.31). With regards to continuous self−reports of chills intensity, results were replicated for Glósóli (mean 95 percent CI = −0.69, −0.61), Jupiter (mean 95 percent CI = −0.73, −0.66), and Ancestral (mean 95 percent CI = −0.79, −0.73), indicating that the chills section appeared to be more emotionally salient than most other moments in the music. A visualization of these simulations can be found in Figure 5.

### 3.6. Psychoacoustic parameters

As a final exploratory process, psychoacoustic parameters were correlated with both phasic SCR and continuous measurements to assess whether some features may be linked to the experience of chills in listeners; given the size of datasets in the correlation computations, and the fact that observations were not independent, statistical significance was ignored, with an emphasis placed on the strength of relationships. The Pearson correlation results (see Table 1) suggest that for Glósóli and Ancestral there are strong positive relationships between continuous measurements of chills intensity and numerous features, namely RMS, brightness, and roughness. Interestingly, for Jupiter, correlations are less consistent and weaker. Furthermore, there appear to be no clear correlations between any psychoacoustic features and phasic skin conductance changes, with some features displaying negative correlations. To account for possible lag between psychoacoustic features and changes in continuous measurements or phasic SCR, cross-correlation analysis was also carried out, which confirmed that the potential lag structure between stimulus qualities and participant responses did not effect the correlation results.

**Table 1:** Pearson correlations between psychoacoustic parameters and both phasic SCR and continuous measures of chills intensity.

	**Continuous measures**			**SCR**		
**Feature**	**Glósóli**	**Jupiter**	**Ancestral**	**Glósóli**	**Jupiter**	**Ancestral**
RMS	0.47	0.56	0.78	−0.49	0.06	0.20
Brightness	0.62	0.13	0.49	−0.09	−0.28	−0.02
Centroid	0.70	0.23	0.61	−0.17	−0.22	0.14
Entropy	0.52	0.18	0.39	−0.30	−0.37	0.03
Roughness	0.80	0.42	0.76	−0.26	0.03	0.17
Flux	0.68	0.50	0.77	−0.40	0.01	0.10
Novelty	0.14	−0.04	0.10	0.11	−0.15	−0.10
Event density	0.31	0.41	0.73	−0.38	0.05	0.17
Key clarity	0.29	0.21	0.25	0.01	−0.39	0.04
Pulse clarity	0.59	−0.08	0.01	−0.24	−0.22	−0.08

## 4. Discussion

The current study provided the first investigation into manipulating musical stimuli and testing effects on the experience of musical chills in an empirical setting, moving beyond the existing limitations of strictly correlative approaches. By removing previously identified chills sections in three pieces of music reported in an earlier survey (Bannister, 2018), the frequency of chills was consistently reduced across listeners and pieces; continuous measurements of chills intensity often decreased significantly, with a similar but less consistent pattern observed for phasic skin conductance activity. It is worth noting that the overall emotional experience during each piece did not significantly change when chills sections were removed, nor were there any clear links between listening conditions and proposed underlying mechanisms of music and emotion (Juslin, 2013), although all three pieces scored highly on rhythmic entrainment and emotional contagion mechanisms; however, chills experiences were seemingly more intense on average when elicited by original versions of the stimuli.

Chills were characterized as an intense and moving response in the experiment when compared to non-chill experiences, although this was largely driven by experiences with Glósóli and Ancestral. Interestingly, both continuous measurements of chills intensity and phasic skin conductance levels generally supported the frequency of chills between conditions, and the emotional salience of chills sections. There were some exceptions and inconsistencies however, particularly with the Jupiter listening conditions, which may be a result of familiarity effects and extra-musical factors.

The results of continuous measurements of chills intensity were clear across Glósóli and Ancestral, indicating that chills sections were emotionally intense compared to musical-control or acoustic-control sections, and removal sections. These findings were less clear in phasic skin conductance; whilst skin conductance was normally significantly higher in chills sections compared with musical-control or acoustic-control sections, this was rarely the case when compared with removal sections. This may firstly be a result of musical qualities in the removal section; for example, the removal section in Glósóli contained a texture and instrument distinct from most of the piece, namely a glockenspiel and “toy box” sound; this sound was often reported as a favorite moment by listeners in the experiment. Furthermore, the Ancestral removal section introduced for the first time a female voice, revisiting lyrics from earlier in the piece, although few participants mention this aspect when discussing favorite moments. However, explanations linked to musical features struggle when considering the continuous measurement results, showing significantly higher scores in chills sections compared to removal sections. A secondary explanation may be related to the experimental design. Some participants heard the original versions of the music first; consequently, when they listen to the same piece again, and are expecting the chills section, some form of veridical expectancy violation may occur when it is removed, referring to explicit expectations formed when one has heard the music before, or is very familiar with the music (Huron and Margulis, 2010; Guo and Koelsch, 2016). This may have confounding effects on physiological markers of emotional arousal such as skin conductance, and may also result in lower continuous ratings if the listener was anticipating a standout or preferred section in the music, only to be disappointed when it is removed. However, this can operate the other way, such that skin conductance results are exaggerated when participants do not explicitly expect the chills section following initial exposure to the manipulated versions of the stimuli. However, every stimulus presentation order was pseudo-randomised and individualised to control for these confounding issues, although small effects may not be fully ruled out. A final explanation may simply be due to the wide variability across individuals with regards to skin conductance (Khalfa et al., 2002); some listeners may be physiologically sensitive and hyper responsive, whereas some may not respond in any way; there may also be habituation effects across time in skin conductance, such that stimulus presentation order may influence this measurement, but less so on continuous measurements. Again however, the pseudo-random presentation orders can control for habituation effects; furthermore, skin conductance measures were normalised within each participant, and mixed-effects linear models incorporate and account for individual differences in reactivity when fitted to the data, so individual sensitivity differences were mostly be captured.

Perhaps a more interesting explanation underlying the negligible difference between chills and removal sections in phasic skin conductance is derived from understanding the impact of local, preceding musical contexts. This is a particularly striking idea given that although the chills section in Glósóli was characterised by a change in dynamics, texture and energy (Sloboda, 1991; Panksepp, 1995; Guhn et al., 2007), and the chills section in Ancestral might be described as the entrance of a new leading instrument and a clear solo and accompaniment relationship (Grewe et al., 2007; Guhn et al., 2007), similar features are also encapsulated in various control sections in these pieces, without resulting in any emotional response similar to those identified in the chills sections. Ancestral provides a notable example, as each control section derived from musicological considerations can be conceptualised either as an entrance of a voice with English lyrics, entrance of a cello over minimal accompaniment, or a pronounced, sudden dynamic and textural change; despite this, the guitar solo remained the most effective elicitor of chills and emotionally intense experiences. This suggests an important role of local musical contexts on the chills potency and emotional salience of musical features within a piece, and this role may be reflected in a relatively small effect of stimulus manipulation on the frequency of chills in the current experiment. This effect of context in musical chills has been alluded to by Panksepp (1995), suggesting that “…it is unlikely that such periods of emotional intensification could have a sufficiently powerful effect to produce chills were it not for the background mood of nostalgic sadness established by the rest of the piece” (p. 193). This should not come as a surprise; a sudden increase in dynamics is only made sudden by preceding musical developments. It may be then, especially when referring to the physiological data, that the moments preceding the onset of the chills sections are also central to the experience and results; regardless of whether listeners heard the chills or removal section, the musical build up to the onset of these epochs may have been an important driver of physiological activity. These considerations align with findings from Salimpoor et al. (2011), noting a distinction in brain activity between anticipatory and experiential phases of the chills response; the notion of a “pre-chill” has also been echoed by Wassiliwizky et al. (2017), and implicates mechanisms of musical expectancy (Huron, 2006; Juslin and Västfjäll, 2008). The relative impact of local musical contexts on the effects of specific musical features or moments linked to chills, currently and in previous literature, appear to be an essential path for future research, and may contribute to ongoing theories of musical tension, release and expectation (Lehne and Koelsch, 2015). However, without attempting to progress from correlative work into causal manipulations of the elusive musical chills response, these interactions and effects cannot be determined.

Even if we consider the possible effects of musical context on the likelihood of musical features eliciting chills, for what reasons do these features induce chills under any circumstances? Research has utilised chills to indicate pleasurable, high arousal emotional states (Blood and Zatorre, 2001; Rickard, 2004; Salimpoor et al., 2009, 2011), and in other studies chills have repeatedly been linked to aspects of musical expectation, such as sudden dynamic changes (Panksepp, 1995; Grewe et al., 2007) and unprepared harmonies (Sloboda, 1991); interestingly, harmonic violations have been found to increase arousal levels in listeners (Steinbeis et al., 2006), and theories have been posited that discuss musical chills in the context of immediate fear responses, followed by slower aesthetic appraisals (Huron, 2006). A different perspective contextualises chills in terms of social separation and closeness (Panksepp, 1995; Panksepp and Bernatzky, 2002), indicated by the mixed state of being moved (Benedek and Kaernbach, 2011; Wassiliwizky et al., 2015), or the communal sharing emotion of Kama Muta (Fiske et al., 2017). In a recent survey on musical chills (Bannister, 2018), it was suggested that due to the variety of psychophysiological and emotional experiences reported during musical chills, and the large corpus of music that elicits the response, the concept of chills may encapsulate several distinct phenomenological experiences that might be defined by the causal underlying mechanisms being activated, such as expectancy violations, fear, awe, or social proximity (see also Maruskin et al., 2012, for examples not specific to music). The current empirical manipulation of musical chills suggests a similar idea: Firstly, emotional experiences with each of the three pieces were characterized by different descriptors, such as happiness and energy for Jupiter, and sadness and tension for Ancestral; secondly, the chills sections across the three pieces appeared to be emotionally salient and linked to chills, resulted in comparable effects when removed, yet shared few similarities with each other. It is possible that these chills sections are engaging with different listening processes (i.e., brain stem reflexes, emotional contagion, rhythmic entrainment, or musical expectancy), that share the physiological outcomes as a commonality, but differ in terms of emotional and aesthetic experiences. If this is indeed the case, an important question would be how specific musical features activate differing mechanisms and result in a multitude of emotional experiences, depending on the local musical structure and context in which they are situated. Following this, the current results may not be fully generalizable across other pieces of music, and this would be predicted considering inconsistencies in previous research; however, this is an outcome of great interest, linked to differing theories and possible conceptualizations of the chills response. It is worth noting that without further systematic investigation this remains conjecture, and it is important to keep in mind what the individual listener and listening context may contribute to the experience.

In terms of psychoacoustic parameters, results show that increases in loudness, brightness and roughness were correlated with increases in continuous measurements of chills intensity in both Glósóli and Ancestral, although few correlations were found with phasic skin conductance. These findings offer some support to previous studies (Grewe et al., 2007), although currently it is not clear as to how these features are linked to chills; perhaps loudness engages a fear response, given that loud sounds normally signify important events; more gradual increases in loudness, such as that in Glósóli, may also allude to an auditory looming effect, and the perception of something approaching oneself (Ghazanfar et al., 2002). Further to this, roughness, linked to dissonance and the critical bandwidths of auditory processing, may be associated with threatening sounds, perhaps from predators, that may elicit fear-induced chills or gooseflesh (Arnal et al., 2015). From a different angle, brightness and higher frequency energy may be a common psychoacoustic attribute across distress vocalizations and music, that which Panksepp (1995) has suggested can result in chills; interestingly, higher pitch has been associated with increased ratings of coldness over warmness in an investigation of audio-tactile metaphors (Eitan and Rothschild, 2010). As it stands, given the lack of research on chills and psychoacoustic parameters, the explanations above are necessarily speculative.

Jupiter perhaps yielded more inconsistent results than the other two pieces in the current experiment, with a more balanced frequency distribution of chills between original and manipulated conditions, slightly less consistent effects of manipulation on continuous measurements, and fewer correlations found with psychoacoustic features. One immediate explanation for these results is that of the three pieces, Jupiter was by far the most familiar to listeners, which introduces a higher possibility of extra-musical effects that could not be controlled, such as the emotional impact of episodic memories (Janata et al., 2007; Janata, 2009; Belfi et al., 2016), or various conditioning effects (Blair and Shimp, 1992; Walther et al., 2005). Familiarity is an important variable to consider, although effects with regards to musical chills are currently inconsistent and unclear (Panksepp, 1995; Rickard, 2004; Benedek and Kaernbach, 2011). A second possibility is that given the longer duration of Jupiter in comparison to other stimuli, and the frequent changes occurring throughout the piece, there is simply more time and scope for the chills response outside of any specific section of music, resulting in a smaller effect when this chills section is removed. It must be noted however that when participants were asked to describe the favorite moment of the piece, many reported exactly the chills section in question, referring to the lyrical adaptation of the theme (“I Vow Thee to My Country”), or the moment when chaotic orchestral movements were calmed by the new string elements and theme. Intriguingly, by removing the chills section, ratings of familiarity were significantly decreased, suggesting that Jupiter could be an interesting candidate for a specific musical progression that is particularly well-known, embedded in a piece that is, generally, less familiar.

There are limitations to the current study worth highlighting. Firstly, the continuous measurements of chills intensity were used as an exploratory method for capturing the nuances and contours of chills experiences that are unlikely to be as simple as experiencing chills or not. However behavioral data from some participants suggest that some listeners used continuous measurements as indicators of general emotional intensity instead; although there is little known about individual thresholds of recognizing chills experiences, some continuous data are consistently reflecting some form of experience, and it is highly unlikely that these listeners are experiencing chills throughout the course of the piece. Furthermore, concurrent tasks during music listening may affect the affective experience (Markovic et al., 2017; Jäncke et al., 2018), although there are some inconsistent results (Hutcherson et al., 2005). Regardless, the continuous measurement task was highly simplified, and whilst some emotional intensity may have been lost, the stimuli remained effective elicitors of chills, and the current results and effects of manipulation were clear. Secondly, as with most chills studies there is a verification problem; although some progress has been made utilizing skin conductance measurements, the response is still only an indicator of general changes in emotional arousal, and despite the use of self-reports, skin conductance and continuous measurements, the chills response has still not been fully confirmed. For future empirical investigations, it is central to assess how chills might better be verified without limiting the possibility of capturing the response in lab settings (see Benedek and Kaernbach, 2011, for an example). Thirdly, the current sample size is quite small; however, it seems unlikely that increasing the size of the sample would affect overall outcomes of this study, and in fact to find robust differences in physiological response and continuous measurements of chills intensity in the current sample is worth noting. In addition, the current sample size is comparable to various studies investigating musical chills (Rickard, 2004; Guhn et al., 2007; Nagel et al., 2008; Egermann et al., 2011). Fourthly, as the manipulation procedure resulted in duration differences between original and manipulated versions of the stimuli, there may be confounding effects of attention or fatigue in the longer versions; however, this was largely accommodated again through stimulus presentation orders, minimal duration differences, and detrending data to deal with longer-term trends resulting from factors such as fatigue. Finally, the current musical manipulations are rather broad, working with larger epochs and sections in music, limiting control over the musical stimuli; however, this was a necessary decision given that manipulations of the chills response are rarely, if ever, investigated in lab settings, and future research should develop on current exploratory findings to target more specific musical or psychoacoustics features in pieces of music.

In conclusion, the current listening experiment demonstrates for the first time the manipulation and suppression of musical chills through changes to various musical stimuli. Results generally converge, showing that predefined chills sections in each piece are judged as emotionally salient and chill-inducing, evidenced through increases in skin conductance, continuous measurements and higher frequency of chills reports in original conditions for all pieces. The chills sections across the three pieces shared few commonalities, and emotional experiences with the three pieces were characterised differently, suggesting that musical chills may be elicited through various listening processes and mechanisms, with each contributing to different emotional experiences that share physiological indices. Furthermore, similar musical features located in other moments of the musical stimuli did not have any comparable effects on emotional experience and chills, suggesting an importance of local, preceding musical contexts that should be systematically investigated in the future. Some important next steps in musical chills research include developing methods that deal with problems of verifying the chills response, understanding the possible differentiation of chills experiences and their links to varying musical features, and the comparison of musical chills with other aesthetic chills responses that occur with films, images and literature, to better understand the variety of aesthetic experiences that are encapsulated by the chills phenomenon.

## Data availability statement

The datasets analyzed for this study can be found at Harvard Dataverse (Suppressing the shills: Self-reports, physiological and psychoacoustic correlates) [https://doi.org/10.7910/DVN/IUCN1Q].

## Author contributions

SB and TE contributed to the conception and design of the study. SB carried out the data collection. SB and TE performed the data analysis. SB wrote the first draft of the manuscript. SB and TE contributed to the manuscript revision, read and approved the submitted version. Early analysis of the current study was presented in a proceedings paper for ESCOM 2017, Ghent, Belgium.

### Conflict of interest statement

The authors declare that the research was conducted in the absence of any commercial or financial relationships that could be construed as a potential conflict of interest.
