# Supplementary material for: Suppressing the Chills: Effects of Musical Manipulation on the Chills Response
Source: Front Psychol. 2018 Oct 29;9:2046. doi: 10.3389/fpsyg.2018.02046 (PMC6215865; doi:10.3389/fpsyg.2018.02046)
Supplement: Supplementary file 1 [file Data_Sheet_1.PDF]

frontiers

Supplementary Material: Suppressing the Chills: Effects of Musical Manipulation on the Chills Response

1 SUPPLEMENTARY DATA

The following supplementary data documentation encapsulates two main aspects: firstly, a supplementary figure (Figure S1) is provided to help visualize the experimental procedure; secondly, an explanation of the pseudorandomization procedure of the stimulus presentation order is provided; finally, a supplementary table (Table S1) is included, which presents descriptive statistics for the sample, and where possible, comparisons between current mean scores and norm population means. The norm scores for musical sophistication were retrieved from Müllensiefen et al. (2014); as we only selected two rating items from the factors of *active engagement, emotion*, and *musical training*, the norm scores were first divided by the total of items attributed to the score, and then multiplied by two. The norm scores for openness were taken from Srivastava et al. (2003), with the single norm score averaged across the norms for ages 20 to 60. Finally, the norms for the Short Test of Musical Preferences were made available at this address: `https://gosling.psy.utexas.edu/scales-weve-developed/short-test-of-music-preferences-stomp/`.

1.1 Procedural Information: Stimulus Presentation

To control for numerous possible confounding variables, such as fatigue, familiarity, and emotional experiences with music relative to previous pieces, the stimulus presentation order was pseudo-randomized across participants, by way of a Latin square formulation. Following this procedure, each participant received a unique presentation order which met the following criteria: No version of a stimulus directly followed or preceded the other version of the same piece; in each block of three stimuli, no more than two of the same manipulation condition were presented (i.e. the two listening blocks always contained a mixture of original and manipulated versions of stimuli).

1.2 Tables

Table S1. Descriptive statistics of the sample and population statistics.

Descriptives		Study Means (SD)	Norm Means
Age		25.20 (5.96)	n/a
Openness		5.19 (0.87)	3.89 (0.68)
**Musical Sophistication**			
	Active Engagement	10.50 (3.55)	9.22 (2.30)
	Musical Training	9.37 (4.83)	7.57 (3.26)
	Emotion	11.25 (2.57)	11.55 (1.68)
**STOMP**			
	Reflective and Complex	4.69 (1.18)	3.87 (1.49
	Intense and Rebellious	4.68 (1.60)	5.00 (1.46)
	Energetic and Rhythmic	4.00 (1.33)	3.99 (1.51)
	Upbeat and Conventional	4.13 (1.16)	3.74 (1.28)

1.3 Figures

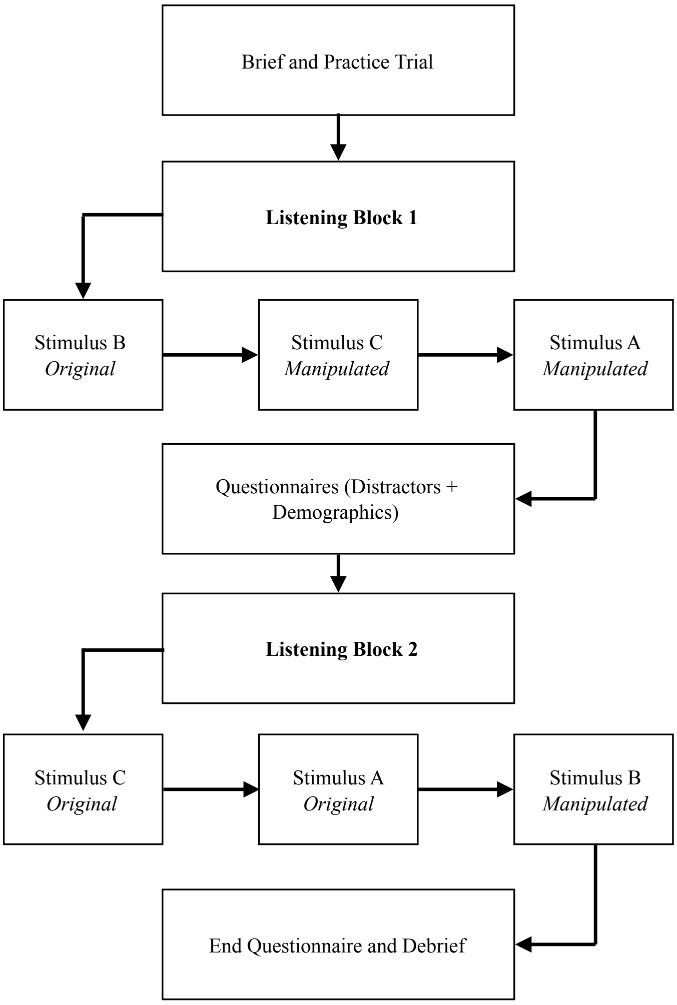

Figure S1. Procedural outline of the listening experiment; note that the stimulus presentation order in the diagram is only one example of pseudo-randomized presentation orders.

REFERENCES

Müllensiefen, D., Gingras, B., Musil, J., and Stewart, L. (2014). The musicality of non-musicians: an index for assessing musical sophistication in the general population. *PloS one* 9, e89642. doi:10.1371/journal.pone.0089642

Srivastava, S., John, O. P., Gosling, S. D., and Potter, J. (2003). Development of personality in early and middle adulthood: Set like plaster or persistent change? *Journal of Personality and Social Psychology* 84, 1041–1053
